# Serum cytokines profile of critically ill COVID-19 patients with cardiac dysfunction

**DOI:** 10.1186/s40635-021-00368-w

**Published:** 2021-01-18

**Authors:** François Bagate, Nicolas Maziers, Sophie Hue, Paul Masi, Armand Mekontso Dessap, Nicolas de Prost

**Affiliations:** 1 Service de Médecine Intensive Réanimation, AP-HP, CHU Henri Mondor, DHU A-TVB, 51, avenue du Mal de Lattre de Tassigny, 94010 Créteil Cedex, France; 2 Faculté de Médecine, Université Paris Est Créteil, Groupe de Recherche Clinique CARMAS, 94010 Créteil, France; 3 Département Immunologie-Hématologie, Hôpitaux Universitaires Henri Mondor, Assistance Publique – Hôpitaux de Paris (AP-HP), Créteil, France; 4 Université Paris-Est Créteil Val de Marne (UPEC), INSERM U95, Créteil, France

To the Editor,

Most patients requiring intensive care unit (ICU) admission for Coronavirus disease 2019 (COVID-19) presented acute respiratory distress syndrome (ARDS) [1]. However, a significant proportion of critically ill COVID-19 patients developed an acute COVID-19 cardiovascular syndrome (ACovCS) characterized by acute myocardial injury, evidenced by an increase in circulating biomarkers including serum troponin and B-type natriuretic peptide, associated with elevated interleukin-6 concentrations, myocardial dysfunction, ventricular arrhythmias, circulatory insufficiency and a high mortality rate [2, 3]. The pathophysiology of ACovCS remains unclear. Infection by the novel severe acute respiratory syndrome coronavirus (SARS-CoV-2) seems to trigger peculiar innate and adaptive immune responses. High concentrations of circulating cytokines involved in the innate immune response [4], have been associated with poor outcomes in COVID-19 patients. We aimed at evaluating whether critically ill COVID-19 patients with cardiac dysfunction exhibited a peculiar immunological phenotype.

Here, we report on clinical and echocardiographic features together with serum levels of cytokines in a monocenter prospective study of critically ill COVID-19 patients admitted to the medical ICU of Henri Mondor Hospital, Créteil, France, between March 8th and March 30th, 2020. The study has received the approbation of an institutional review board (Comité de Protection des Personnes Ile de France II; reference number: 3675-NI). Informed consent was obtained from all patients or their relatives. Reverse-transcriptase polymerase chain reaction (RT-PCR) assays of nasopharyngeal swabs were positive for SARS-CoV-2 in all patients. Blood samples were drawn within 48 h of ICU admission to measure cytokines concentrations using Luminex® multiplex bead-based technology (R&D Systems, Minneapolis, MN, USA) on serum diluted ½. Echocardiographies were performed within the same time frame to characterize cardiac dysfunction (see supplemental method section) and categorize patients according to LVEF tertiles. Patients were not receiving dobutamine at the time the first echocardiography was performed, except for one patient. Principal component analysis (PCA) was used to summarize cytokines profiles information and assess its relation with LVEF tertiles (see Additional file 1 for a detailed description of the statistical methods).

Additional file 2: Table S1 shows the characteristics of the 34 consecutive patients included in this study cohort, four of whom had previously known moderate LVEF dysfunction (i.e., between 35 and 50%). The first plan of the PCA accounted for 46.2% of inertia, thus almost half the total variance or inertia, and was mainly driven, among the 19 serum cytokine assays, by three of them, involved in the innate immune response (i.e., IL-10, GM-CSF and CXCL10/IP-10), which were highly correlated between each other and thus formed a coherent cytokine group (Fig. 1a). Moreover, this data partitioning built on the immunological phenotyping at ICU admission was significantly, and independently, connected to LVEF tertiles categorization (Fig. 1b) (tertile 1: hypokinetic patients, LVEF between 28 and 55%; tertile 2: normokinetic patients, LVEF 55–67%; tertile 3: hyperkinetic patients, LVEF 67–80%), indicating that patients with different values of LVEF had contrasted serum cytokines profiles.

**Fig. 1:**
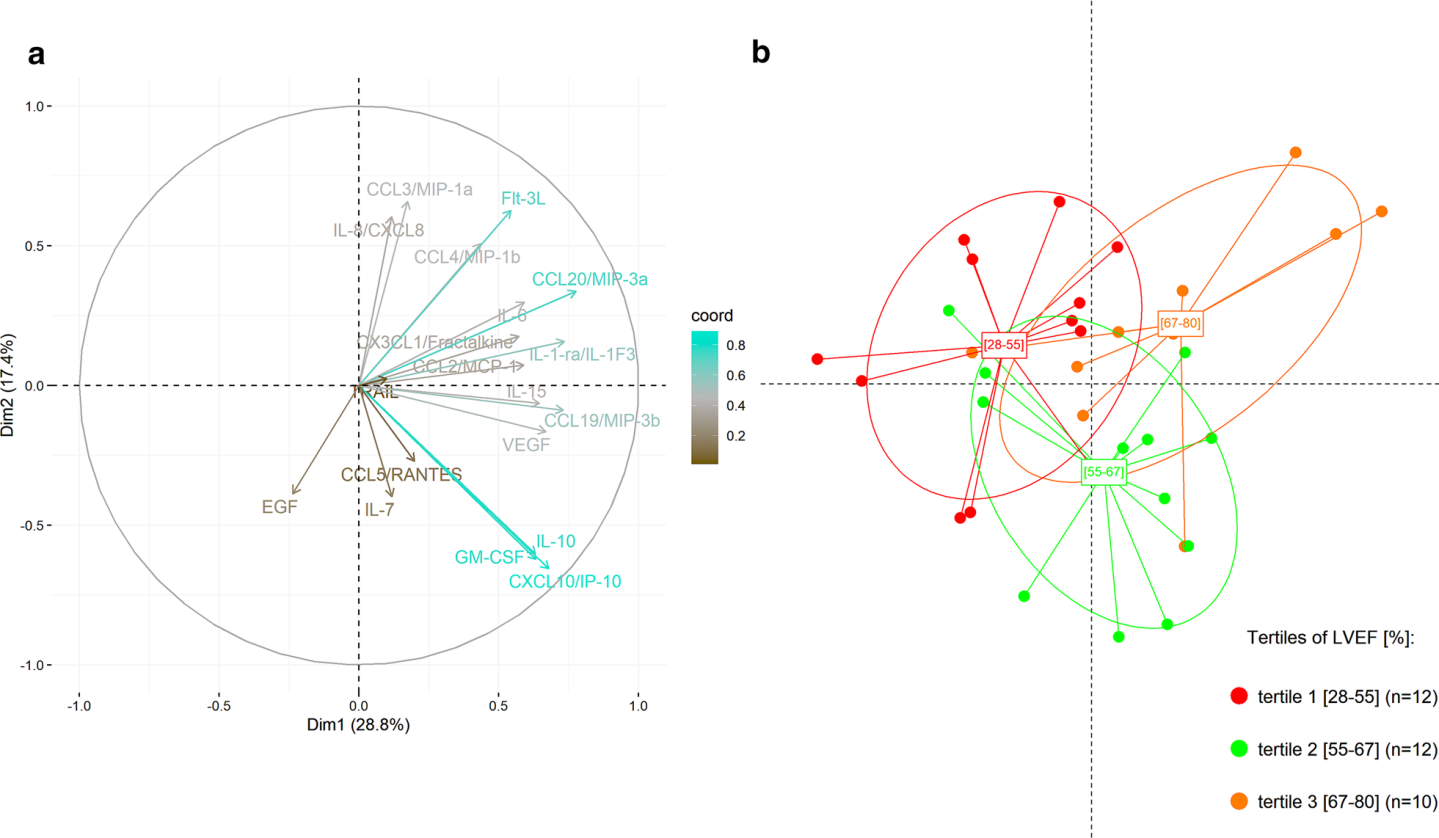
Immunological phenotyping principal component analysis (PCA) linked to left ventricle ejection fraction (LVEF) tertiles categorization. **a** PCA of the 19 cytokines variables obtained from blood sampled of 34 COVID-19 ICU patients. Displayed as graph of the variables, correlation circle is represented around the first plan PCA: the first axis (Dim1) being orthogonal to the second axis (Dim2). Each vector (arrow) of these 19 original variables was gradient-colored related to its coordinate (coord) values on these two axes. **b** immunological phenotyping, PCA-derived latent variables, in connection with LVEF categorized by tertiles. Displayed as graph of the individuals between-class analysis (bca) of PCA was calculated from the same 19 variables in the sampled patients (*n* = 34). So dually, the diagram depicted the first principal plan represented by 28% and 17% of whole inertia for axis one (horizontal) and second (vertical) axis, respectively. The between-class inertia percentage was 11% (*p* = 0.022). Categorization in tertile 1 [28–55] (*n* = 12), tertile 2 [55–67] (*n* = 12), and tertile 3 [67–80] (*n* = 10) of LVEF [%] was used as the instrumental variable and colored-labeled for each patient individual (point). The observed probability resulted from a Monte Carlo permutation test done on this between-class inertia

We herein showed that there were three distinct serum cytokines patterns according to cardiac function, as assessed by LVEF, in critically ill COVID-19 patients. Interestingly, LVEF tertiles were also significantly associated with blood troponin levels (Additional file 3: Fig S1), highlighting a global association between serum cytokines profiles, cardiac dysfunction and troponin elevation. The finding of an IP-10, IL-10, and GM-CSF signature highlights the contribution of myeloid cells to pathogenic inflammation, as previously reported [5]. Such inflammation may amplify an auto-inflammatory loop leading not only to lung, but also myocardial, injury. Further studies, including myocardial tissue and cardiac magnetic resonance studies, are needed to assess the contribution of associated acute myocarditis lesions. Our study highlights the potentially pathogenic association between serum cytokines profiles and myocardial injury in critically ill COVID-19 patients. However, because up to 29% of patients with severe SARS-CoV-2 infection and cardiac dysfunction have a history of coronary heart disease [6], we cannot exclude that a substantial proportion of our patients had a previously unknown heart failure. Our study has a number of limitations related to the small number of patients included and the lack of a non-COVID-19 control group, making the results only exploratory. In conclusion, our results establish a link between serum cytokines profiles and LVEF in patients with severe SARS-CoV-2 infection, but do not allow for causal inferences to be drawn regarding the mechanisms at play.

## Supplementary Information

**Additional file 1:** Supplemental methods.

**Additional file 2: Table S1.** Clinical characteristics of 34 critically ill COVID-19 patients, according to left ventricle ejection fraction (LVEF) tertiles.

**Additional file 3: Figure S1.** Serum troponin concentrations as a function of tertiles of left ventricle ejection fraction (LVEF) in critically ill COVID-19 patients.

## Data Availability

All data generated and analyzed during the study are included in the published article and can be shared upon request. All authors helped to revise the draft of the manuscript. All authors read and approved the final manuscript. The study has received the approbation of an institutional review board (Comité de Protection des Personnes Ile de France II; reference number: 3675-NI). Informed consent was obtained from all patients or their relatives.

## Abbreviations

ICU: Intensive care units
COVID-19: Coronavirus disease 2019
ARDS: Acute respiratory distress syndrome
ACovCS: Acute COVID-19 cardiovascular syndrome
SARS-CoV-2: Severe acute respiratory syndrome coronavirus 2
LVEF: Left ventricle ejection fraction
RT-PCR: Reverse-transcriptase polymerase chain reaction
PCA: Principal component analysis
