# Supplementary material for: Serum cytokines profile of critically ill COVID-19 patients with cardiac dysfunction
Source: Intensive Care Med Exp. 2021 Jan 18;9:2. doi: 10.1186/s40635-021-00368-w (PMC7812556; doi:10.1186/s40635-021-00368-w)
Supplement: Supplementary file 1 — Additional file 1: Supplemental methods. [file 40635_2021_368_MOESM1_ESM.docx]

**Serum cytokines profile of critically ill COVID-19 patients with cardiac dysfunction**

**Supplementary file 1**

**Methods**

*Measurements of serum cytokine concentrations*

Blood samples collected at days 1-2 were immediately centrifuged for storage at – 80°C. Cytokines were measured at distance in serum inactivated for 20 minutes at 56°C using Luminex® multiplex bead-based technology (R&D Systems, Minneapolis, MN. USA) and a Bio-Plex 200® instrument (BioRad, Hercules, CA. USA), according to the manufacturers’ protocols on serum diluted to 1/2. Laboratory technician who performed the assays was blinded to all clinical data. The following cytokines/chemokines were evaluated CCL2/MCP-1, CCL4/MIP-1β, CCL19/MIP-3β, CD40L, CXCL10/IP-10, FGF-basic, G-CSF, GRZ-B, IFN-β, IL-1α, IL-1ra, IL-3, IL-5, IL-7, IL-10, IL-13, IL-17A, PD-L1, TNF-α, VEGF, CCL3/MIP-1α, CCL11/Eotaxin, CCL20/MIP-3α, CX3CL1/Fractalkine, EGF, Flt-3L, GM-CSF, IFN-α, IFN-γ, IL-1β, IL-2, IL-4, IL-6, IL-8/CXCL8, IL-12p70, IL-15, IL-33, TGF-α, TRAIL, CCL5/RANTES, IL-17E/IL-25. All samples were inactivated during 20 minutes at 56°C, as previously described (4). We analyzed cytokines/chemokines which displayed more than 80% of concentration values above the lower limit of quantification (n=19). For each of these analytes, extrapolated concentration values calculated by Bioplex Manager 6.1 software were taken into account and undetectable values were imputed to the lowest extrapolated concentration value.

*Statistical analysis*

As all the questions formulated in this study had been placed in a purely exploratory objective, the exclusive purpose of our analysis was descriptive. The "classical" significance threshold of 0.05 for p values was used. All statistical tests were two-sided. All analyses were performed in R [1] (version 4.0.2, Vienna, Austria). As and when they were used, we mentioned the R packages that we employed as well as some of their respective functions (in italic). For bivariate exploration, non-parametric tests were exclusively employed. Wilcoxon rank sum test (Wilcoxon signed rank test), or Kruskal-Wallis rank sum test where appropriate, was carried out when confronting a numerical variable with a categorical variable. To study the independence of two categorical variables, Fisher's exact test was used. The multivariate ordination analysis was performed exclusively with the log10 transformed table of the 19 immunological variables. After that, once these variables have been preprocessed by centering and scaling, principal component analysis (PCA) was applied. To present multivariate analysis results, the first factorial plan, by definition the one with the highest amount of information (expressed as percentage of inertia for each axis), was exhibited with Its first (horizontal) and second (vertical) axis drawn in dotted lines. The graph of variables or correlation circle was drawn with *fviz_pca_var* function with col.var = "coord". Indeed, with this last specification, the colors for variables were automatically controlled by their coordinates (x² + y²). We chose the latter in so far as it was a kind of balance with quality of representation and contribution of each variable for the factorial plan. For the representation of the graph of individuals and in order to reveal at best the inter-group total variance or inertia, between-class analysis (bca) was then applied to pca results. The associated p value resulted from a Monte-Carlo permutation test (*randtest* function with nrepet = 999 and alter = "two-sided" arguments) on this between class inertia (in percentages). In the graph of individuals, each of plotted point, with their coordinates, was also colored according to its categorical belonging: the instrumental variable in the case of the between-class analysis. While the dots seemed to outline a singular shape for each colored cloud, all individual points were joint to the label of their respective belonging, which was, for each category, positioned at barycenter. All functions used for multi-dimensional exploration, with their statistical or graphical outputs, were implementations from ade4 [2], from FactoMineR [3] or from factoextra R packages.

**References**

[1] R Core Team. R: A Language and Environment for Statistical Computing. (2020). Available at: <https://www.r-project.org/>.

[2] Dufour, A.-B. & Stéphane Dray. The ade4 Package: Implementing the Duality Diagram for Ecologists. *J. Stat. Softw.* **22**, (2007).

[3] Lê, S., Josse, J. & Husson, F. FactoMineR: A Package for Multivariate Analysis. *J. Stat. Softw.* **25**, 1–18 (2008).
