# Supplementary material for: Serum cytokines profile of critically ill COVID-19 patients with cardiac dysfunction
Source: Intensive Care Med Exp. 2021 Jan 18;9:2. doi: 10.1186/s40635-021-00368-w (PMC7812556; doi:10.1186/s40635-021-00368-w)
Supplement: Supplementary file 2 — Additional file 2: Table S1. Clinical characteristics of 34 critically ill COVID-19 patients, according to left ventricle ejection fraction (LVEF) tertiles. [file 40635_2021_368_MOESM2_ESM.docx]

| **Supplementary Table 1.** Clinical characteristics of 34 critically ill COVID-19 patients, according to left ventricle ejection fraction (LVEF) tertiles. | | | | | |
| --- | --- | --- | --- | --- | --- |
| **Variables** | **All**  **N=34** | **Tertile 1 [28-55]***  **N=12** | **Tertile 2 [55-67]***  **N=12** | **Tertile 3 [67-80]***  **N=10** | **P value** |
| Age (years) | 63 (51-73) | 70 (62-73) | 54 (45-71) | 64 (44-74) | 0.21 |
| Male gender | 29 (85%) | 10 (83%) | 9 (75%) | 10 (100%) | 0.25 |
| BMI (kg/m^2^) | 28 (26-32) | 30 (26-32) | 29 (26-35) | 26 (23-28) | 0.04 |
| ***Comorbidities*** |  |  |  |  |  |
| Current smoker | 14 (41%) | 8 (67%) | 2 (17%) | 4 (40%) | 0.05 |
| Arterial hypertension | 17 (50%) | 7 (58%) | 5 (42%) | 5 (50%) | 0.72 |
| Diabetes mellitus | 10 (29%) | 4 (33%) | 2 (17%) | 4 (40%) | 0.46 |
| COPD | 5 (15%) | 4 (33%) | 0 | 1 (10%) | 0.06 |
| Chronic Dialysis | 1 (3%) | 0 | 1 (8%) | 0 | 0.39 |
| Chronic heart failure | 6 (18%) | 4 (33%) | 2 (17%) | 0 | 0.12 |
| ***Organ failure and outcomes*** |  |  |  |  |  |
| SAPS-II at admission | 38 (31-44) | 42 (38-82) | 34 (30-40) | 36 (29-54) | 0.04 |
| SOFA score at admission | 9 (6-10) | 9 (6-10) | 7 (6-9) | 8 (5-10) | 0.43 |
| Hydroxychloroquine use | 19 (59%) | 6 (50%) | 8 (67%) | 5 (50%) | 0.65 |
| PaO_2_/FiO_2_ (mmHg) | 132 (100-180) | 132 (88-158) | 171 (118-194) | 112 (88-142) | 0.18 |
| NMBA use | 34 (100%) | 12 (100%) | 12 (100%) | 10 (100%) | >0.99 |
| Prone positioning | 32 (94%) | 11 (92%) | 11 (92%) | 10 (100%) | 0.64 |
| ARDS | 34 (100%) | 12 (100%) | 12 (100%) | 10 (100%) | >0.99 |
| Total PEEP (cmH_2_O) | 11 (9-12) | 12 (10-14) | 11 (8-12) | 12 (9-12) | 0.40 |
| Driving pressure | 12 (11-15) | 11 (10-12) | 14 (12-14) | 14 (11-16) | 0.05 |
| Crs (mL/cmH_2_O) | 34 (26-42) | 36 (31-44) | 32 (28-35) | 31 (25-44) | 0.27 |
| RRT | 18 (53%) | 8 (67%) | 6 (50%) | 4 (40%) | 0.44 |
| LVEF (%) | 62 (53-70) | 44 (34-54) | 63 (61-64) | 72 (70-77) | <0.01 |
| Serum troponin (ng/L) | 20 (11-41) | 40 (15-264) | 15 (6-40) | 13 (9-27) | 0.02 |
| Serum Lactate (mmol/L) | 1.5 (1.2-1.9) | 1.5 (1.2-2.0) | 1.7 (1.3-1.9) | 1.4 (1.0-2.1) | 0.90 |
| Shock (vasopressor use) | 20 (59%) | 8 (67%) | 6 (50%) | 6 (60%) | 0.71 |
| CI (L/min/m^2^) | 3.1 (2.6-3.4) | 2.8 (2.1-3.8) | 3.2 (2.9-3.6) | 3.0 (2.6-3.3) | 0.38 |
| Inotrope use | 5 (15%) | 5 (42%) | 0 | 0 | <0.01 |
| Veno-venous ECMO | 9 (27%) | 3 (25%) | 4 (33%) | 2 (20%) | 0.77 |
| Day-28 mortality | 11 (32%) | 6 (50%) | 3 (25%) | 2 (20%) | 0.26 |
| * Values between brackets indicate minimal and maximal LVEF values for each tertile; Continuous variables are expressed as median (inter-quartile range 25-75). COVID-19: coronavirus disease 2019, BMI: body mass index, COPD: chronic obstructive pulmonary disease, SAPS II: Simplified Acute Physiology Score II, SOFA score: Sequential Organ Failure Assessment, PaO_2_: arterial oxygen tension, FiO_2_: fraction of inspired oxygen, NMBA: neuromuscular blocking agent, ARDS: acute respiratory distress syndrome, PEEP: positive end expiratory pressure, Crs: Respiratory system compliance, Total PEEP and plateau pressure were measured by short end-expiratory and end-inspiratory occlusions, respectively, RRT: renal-replacement therapy, LVEF: left ventricle ejection fraction, CI: cardiac index, ECMO: extracorporeal membrane oxygenation. | | | | | |
